# Integrated evaluation of antifungal activity of pomegranate peel polyphenols against a diverse range of postharvest fruit pathogens

**DOI:** 10.1186/s40643-025-00874-9

**Published:** 2025-04-15

**Authors:** Muhammad Nawaz, Junkun Pan, Hui Liu, Muhammad Jawad Umer, Jiechao Liu, Wenbo Yang, Zhenzhen Lv, Qiang Zhang, Zhonggao Jiao

**Affiliations:** 1 Zhengzhou Fruit Research Institute, Chinese Academy of Agricultural Sciences, Zhengzhou, 450009 Henan China; 2 Zhongyuan Research Center, Chinese Academy of Agricultural Sciences, Xinxiang, Henan, 453000 Henan China; 3 Guangdong Provincial Key Laboratory of Crop Genetic Improvement, Peanut Sub-Center of National Center of Oilseed Crops Improvement, Crops Research Institute, Guangdong Academy of Agricultural Sciences, 510640 Guangzhou, Guangdong, South China China

**Keywords:** Pomegranate peel, Polyphenols, Antifungal activity, Fruit, Postharvest pathogens

## Abstract

The search for safe, natural antimicrobial agents has received significant attention for controlling postharvest diseases in fruits. This study evaluated the antifungal activity of pomegranate peel extracts (PPE) against 9 pathogenic fungi by different methods. Additionally, the key antifungal polyphenol compounds were identified. Results revealed that the n-hexane partitioned fraction of ethanolic extract exhibited the highest inhibition efficacy. A total 36 polyphenols, including 10 newly discovered compounds in pomegranate peel were recognized. Among these, 9 specific polyphenols were found to be relatively abundant in the n-hexane fraction, highlighting their potential as primary antifungal agents. Notably, the newly identified polyphenol compound nobiletin demonstrated the strongest inhibitory effects against *Colletotrichum gloeosporioides*, *Rhizopus stolonifer* and *Aspergillus niger* with inhibition diameters of 12.2, 12.0, and 12.5 mm, respectively. Salidroside exhibited significant antifungal activity against *Monilinia fructicola* and *A. niger* (inhibition diameters > 12.0 mm). Furthermore, cinnamic acid that was enriched in hexane fraction, showed the highest inhibitory zones against *Alternaria alternata*, *C. gloeosporioides* and *Botryosphaeria dothidea* (11.8 ~ 12.7 mm). Caffeic acid and protocatechuic acid both displayed the lowest minimum inhibitory concentration (MIC) on *Penicillium expansum*. The study demonstrated the potential of PPE, particularly the n-hexane fraction, as a broad-spectrum natural antifungal agent for postharvest disease management.

**Supplementary Information:**

The online version contains supplementary material available at 10.1186/s40643-025-00874-9.

## Introduction

Multiple biotic and abiotic factors are responsible for postharvest losses. It is estimated that postharvest decay deduced by fungi in fruits is thought to be more than 25% in developed countries (Nunes 2012). This rot causes mycotoxin contamination and reduction of quality, market value, and nutrient composition of fruits (Gomes et al. 2015). It has been reported that numerous fungi belonging to various genera were associated with the postharvest rot of fruits. Among these, *Colletotrichum gloeosporioides* and *Botrytis cinerea*, were considered the most dangerous pathogens affecting several plant hosts (Zhao et al. 2020). *Aspergillus niger* (Kumar et al. 2016), *Alternaria alternata* (Garganese et al. 2019) and *Botryosphaeria dothidea* (Park et al. 2023) were also notable pathogens that caused postharvest rots. *Aspergillus flavus* is a prominent fungus renowned for producing mycotoxins and causing postharvest rot (Velazhahan 2023). *Rhizopus stolonifer* is a well-known fungal pathogen of peaches and is responsible for Rhizopus rot, which is also known as soft rot (Bautista-Baños et al. 2014). Genus *Monilinia* is the necrotrophic pathogens to infects the stone fruits (Obi et al. 2018). Additionally, many *Penicillium* species such as *Penicillium digitatum*,* P. italicum and P. expansum*, also posed significant threats to many economically important fruits (Zhang et al. 2020).

Even though the postharvest fungal diseases of fruits can be controlled by synthetic fungicides, their high doses may cause many problems, for example, high or low residual toxicity, human health problems, pathogens resistance, environmental pollution and carcinogenicity in humans (Kahramanoğlu 2024). Additionally, due to the toxicological effects of synthetic fungicides, inorganic compounds registered for the controlling of postharvest fungal diseases were very limited and the consumer demand for food that was free of fungicides were also increasing (Lastochkina et al. 2020). This trend’s requirements called for the production of alternative methods that were based on natural products derived from plants that are biodegradable, non-toxic, safe and eco-friendly (Zheng et al. 2019). There was a surge in research interest in the search for natural antifungals, particularly plant-derived compounds, as a substitute for synthetic fungicide products for fruit preservation.

Pomegranate (*Punica granatum*) is a fruit that grows in many countries with numerous potential benefits as food and medicine (Gosset-Erard et al. 2021). It has many types of primary and secondary metabolites with antihypertensive, antiulcerogenic, antidiabetic, anti-obesity, anticancer, and antimicrobial activities (Akhtar et al. 2015). The beneficial properties are not only limited to the edible part of the fruit but the non-edible parts of the plant such as leaves, buds, bark, flowers, peel and seeds also possess these activities (Orgil et al. 2014). The peel of pomegranates is an important part of the fruit, contributes almost 50% of its total weight and is an important source of bioactive compounds (Elkahoui et al. 2024; Salim et al. 2023; Jebahi et al. 2022; Sreekumar et al. 2014; Viuda-Martos et al. 2010). The major bioactive compounds present in the peels were polyphenols and polysaccharides, of which the polyphenols showed a wide range of biological activities, such as anti-inflammatory, antiviral, antioxidant, anti-cancer, antioxidant, and so on (Deng et al. 2017; Salim et al. 2023). It was also reported that these compounds were effective against the fungus pathogens (Tehranifar et al. 2011; Foss et al. 2014). Therefore, the pomegranate peels can be used as bioactive ingredients to effectively utilize the by-product resources and contribute positively to the pomegranate industry.

Several studies have demonstrated the antifungal activity of pomegranate peel extract (PPE) against postharvest fruit pathogens, including *B. cinerea*, *P. digitatum*, *P. expansum* (Nicosia et al. 2016) and *A. niger* (Ibrahium 2010). These studies have highlighted PPE’s potential as a natural alternative for controlling fruit postharvest diseases due to its bioactive compounds, particularly polyphenols. Despite these promising findings, most research has primarily focused on the pomegranate peel extract effects on a narrow range of fungal species and its general antifungal properties. Additionally, only a few major polyphenols in pomegranate peel, such as punicalin, punicalagin, gallic acid and ellagic acid have been investigated regarding the antifungal activity against postharvest fungi (Brighenti et al. 2021). This narrow scope highlights the need for more comprehensive studies to explore the full spectrum of antifungal compounds in pomegranate peel. Besides, the antifungal profiles of pomegranate peel polyphenols on different fungi remained underexplored. This study aims to address these gaps by identifying key antifungal compounds in PPE and evaluating their antifungal efficacy. In the present work, a diverse range of fungi associated with postharvest fruit rot were used to evaluate the antifungal activity of pomegranate peel extract and its key polyphenol compounds. Especially, to the best of our knowledge, this is the first report on the antifungal activity of pomegranate peel extract against *B. dothidea and Monilinia fructicola.* This research will enhance the knowledge of the antifungal properties of pomegranate peel and provide valuable insights for the development of natural fungicides to reduce postharvest losses in fruits.

## Materials and methods

### Plant materials

The mature fruits of the *Punica granatum* (Tunisia cultivar) were obtained from a local orchard in Xingyang, Henan province, China. The pomegranate fruits were washed with running tap water. The carpelar and aril membranes were removed from the fruit peels. The peels were cut into small pieces and shade dried for 2 weeks. The dried peels were stored in the cold storage house for further use.

### Preparation of pomegranate Peel extracts by different solutions

**Fig. 1:**
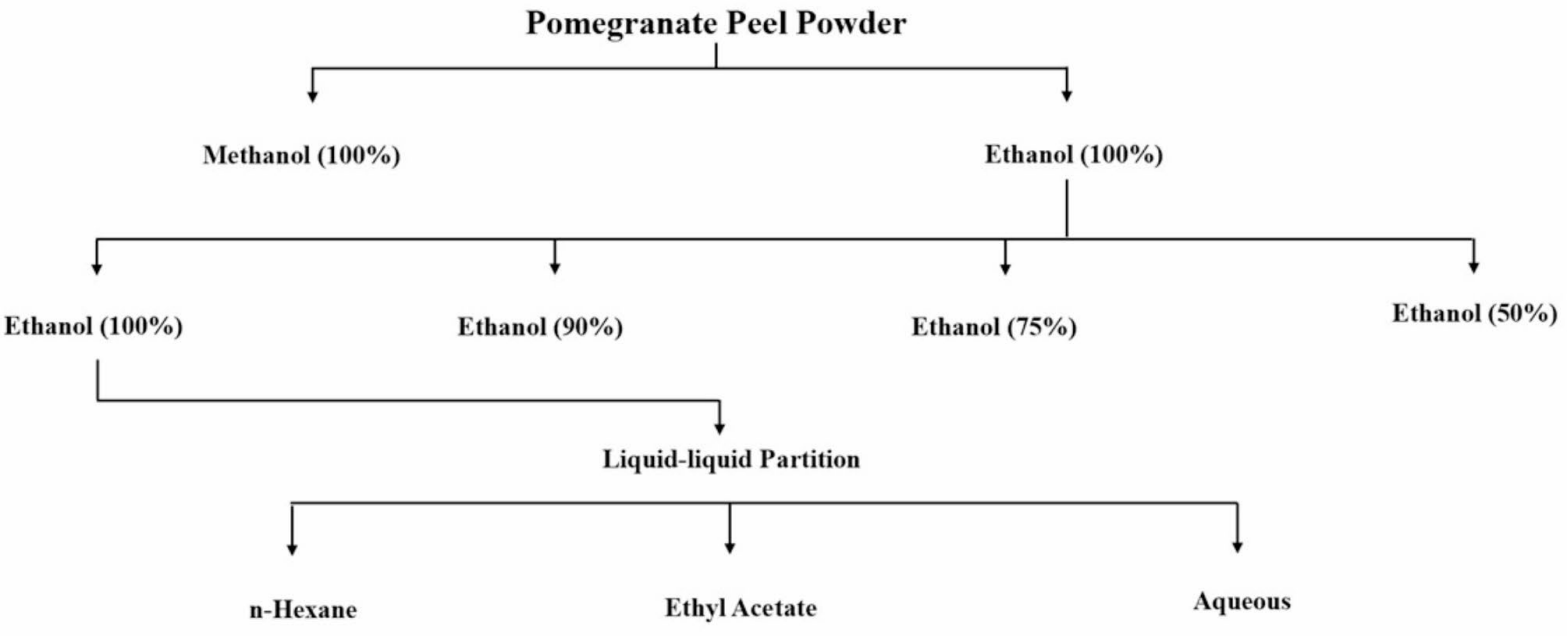
Extraction procedure of pomegranate peel components using solvent partitioning

The preparation of different pomegranate peel extracts is shown in Fig. 1. The dry pomegranate peel (PP) was ground and 100 g of the powder was macerated with 300 mL of methanol or ethanol (100%) and shaken in a dark incubator at room temperature for 24 h. After that, the solutions were filtered using Whatman filter paper (No. 1), and the residue was extracted twice. Then the supernatants were coalesced and evaporated in vacuum at 35 °C using a rotary evaporator. The residues obtained were frozen at -80 °C for 24 h and dehydrated in a vacuum freeze dryer (ALPHA 2–4 LSCplus, CHRIST, Germany). During the extraction that followed, 4 different concentrations (100%, 90%, 75% and 50%) were used to compare the different ethanol crude extracts by the above-mentioned process.

### Liquid-liquid partition of the pomegranate Peel extract

The 100% ethanol crude extract that showed the highest antifungal activity was used for the liquid-liquid partition by using the different solvents with increasing polarity following the method of Rosas-Burgos et al. (2009) with some modifications. The crude extract was suspended in 50 mL of distilled water and fractions were made by adding solvents (150 mL, twice) successively with increasing polarity i.e., n-hexane, ethyl acetate and shaken vigorously. The layers were separated accordingly and the soluble remainder was used as a residual aqueous fraction. The 3 partitioned fractions (n-hexane, ethyl acetate, and aqueous) were rotary evaporated and vacuum freeze dried according to the above-mentioned method. The schematic representation of pomegranate peel extraction via solvent partitioning was graphically depicted in Fig. 1.

### Method for cultivating and Preparing of fungus inoculum

In this study, 9 different postharvest fungi of fruits were used. *R. stolonifer*,* A. niger*,* and A. flavus* fungal strains were isolated from decayed fruits, among them the *R. stolonifer* was isolated from peach, *A. niger* from blueberry and *(A) flavus* from strawberry. These 3 fungal strains were identified through morphological, microscopic and molecular identification. *C. gloeosporioides*,* B. dothidea*, and *B. cinerea* were taken from the Postharvest Disease Lab, Research Institute of Pomology, CAAS. *A. alternata* (ACCC 36135), *M. fructicola* (ACCC 36262), and *P. expansum* (ACCC 30904) fungi strains were bought from the Agricultural Culture Collection of China (ACCC). These fungi strains except *B. dothidea* were grown on potato-dextrose agar plates for 1 to 3 weeks at 25 ± 2 °C in the incubator. Malt extract agar (2%) was used for the culture of *B. dothidea* by applying an irregular 12-h cycle of near-black UV light to induce spores (Wang et al. 2021). To recover the fungus spores, sterile double distilled water was added to the fungus agar plate surface, followed by a gentle scraping using a sterile L-shaped plastic rod to collect the maximum number of spores. The spore’s suspension was filtered by using the sterile muslin cloth and vortexed for 60 s to obtain a uniform mixture. The number of spores in the suspension was determined by using a hemocytometer expressed as the number of spores per millilitre (spores/mL). The spore suspensions were serially diluted to obtain approximately 1 × 10^7^ spores/mL.

### Evaluation of the antifungal activity of pomegranate peel extracts (PPE) and its fractions by agar well diffusion method

The agar well diffusion method was used to evaluate the antifungal activity of extracts and fractions of pomegranate peel (Gonelimali et al. 2018). For that, 1 mL of freshly prepared spore suspensions of 9 postharvest fungi were pipetted into the centre of sterile petri plates (90 mm in diameter). Cooled molten Sabouraud Dextrose Agar (SDA) is poured into the petri plates containing the fungi spores and mixed well with the help of an L-shaped plastic rod. Keep it for 30 min, upon solidification, wells were made into the agar plates containing fungus inoculum with the help of a sterile cork borer (8 mm). After that, 100 µL of each solvent extract (dissolve 70 mg of each dry extract into 1 mL of 10% DMSO) was added into the respective holes. DMSO (10%) was taken as a negative control. The plates were kept for 30 min so that the crude extracts were diffused into the agar. Then these plates were placed into the incubator at 25 ± 2 °C. After 3 days, the zone of inhibition (including well diameter) was measured with the help of a Vernier caliper.

### Determination of total polyphenol contents (TPC) and total flavonoid contents (TFC) in pomegranate Peel fractions

The pomegranate peel extracts (0.1 g) were dissolved into 20 mL of methanol and then ultrasonic for 30 min at 40 °C. After that, the mixtures were centrifuged at 4000 r/min for 10 min and were used for the estimation of total polyphenol and flavonoid contents (Liu et al. 2023). Total polyphenol contents in the PPE were determined by using the method of (Lv et al. 2024) with slight modification. Briefly, take 100 µL of each supernatant of PPE and dissolve it into the 1 mL of Folin–Ciocalteu reagent (1:10, v/v), and 3 mL of sodium carbonate (75 g/L). The reaction mixtures were kept in the dark for 2 h. The absorbance was measured at 765 nm and gallic acid was used as standard.

Total flavonoid content in the peel extracts was determined by the method described by (Liu et al. 2019) with minor modifications. Briefly, take 100 µL of each supernatant of PPE and dissolve it into the 0.3 mL of (5%, w/v) sodium nitrite. Keep the solution at room temperature for 8 min. Then 0.3 mL of aluminium nitrate (10%, w/v) was added to the reaction mixture and kept for 8 min. Afterward, 4 mL of (1 M) sodium hydroxide was added. The absorbance was measured at 510 nm after 15 min. Rutin was used as a standard.

### Recognition of polyphenols by ultra-performance liquid chromatography coupled with quadrupole time-of-flight mass spectrometry (UPLC-Q-TOF/MS)

For the recognition of polyphenol compounds in the n-hexane, ethyl acetate and water fractions, firstly 6 mg of these fractions was dissolved into 1 mL of methanol. All samples were filtered through a 0.22 μm filter before being used for LC/MS and HPLC analysis. To determine the presence of polyphenols in these fraction solutions, the LC-Q-TOF/MS system (UPLC, Waters, Iclass, Wilford, USA; MS, AB Sciex, Triple TOF 4600, Framingham, MA, USA) was used according to the specified method already reported by Yang et al. (2023) with some modifications. To separate the polyphenols, a BEH C18 column (2.1 × 100 mm,1.7 μm) (Waters Co., Milford, MA) was used. The mobile phase included acetonitrile (solvent A) and ultrapure water (solvent B). The column temperature was 35 °C, mobile phase was delivered at a flow rate of 0.25 mL/min and the injected volume was 2 µL with sample detection occurring at a wavelength of 290 nm. The gradient elution was set as follows: 5% A at 0 min; 8% A at 3 min; 10% A at 6 min; 30% A at 15 min; 60% A at 21 min; 80% A at 23 min; 100% A at 25 min; 100% A at 27 min; 5% A at 28 min; 5% A at 30 min.

The MS was worked at ESI (electrospray ionization) mode with negative and positive ions. The optimal conditions were as follows: the ion spray voltage for positive mode was set to 5500 V, and for negative mode, it was − 4500 V; the temperature was kept at 550 °C; the curtain gas was 35 psi; ion GS1 (source gas 1) was set at 50 psi; the ion source gas 2 (GS2) was also adjusted to 50 psi; for the positive mode, the declustering potential was 100 V, while for the negative mode, it was − 100 V. The positive mode’s scan range of TOF masses was around 50–1200 Da, whereas the negative mode’s range was 65-1200 Da. The instrument’s mass accuracy was instantly corrected using the APCI positive/negative calibration solution. Polyphenols were recognized by cross-referencing them with the Metabolites database and TCM MS/MS library.

### Quantification of the key polyphenol compounds in the n-hexane fraction of pomegranate peel extract by HPLC

For the quantification of the key polyphenol compounds, the e2695 HPLC system was used by following the method of Yang et al. (2023) with some modifications. The analysis was carried out at 280 nm UV wavelength with a UV-2489 detector by using the HPLC Waters Symmetry C18 column (250 mm ×4.6 mm, 5 μm). Solvent A (acetonitrile) and solvent B (2 mL of acetic acid in 998 mL of water) were served as mobile phases. The solvent gradient program was set as follows: 5% A, 0.01 min; 17% A, 11 min; 18% A, 13 min; 27% A, 20 min; 30% A, 23 min; 40% A, 30 min; 100% A, 33 min; 5% A, 38 min; 5% A, 39 min. The mobile phase was delivered at a flow rate of 1 mL/min and the injected volume was 10 µL. The results of polyphenol compounds in the sample are expressed as mg/g.

### Evaluation of antifungal activity of key polyphenol compounds by disc diffusion method

The antifungal activity of identified key polyphenol compounds was evaluated against the postharvest fungi by the disc diffusion method as described by Dikbas et al. (2008) with minor modifications. Fungus spore’s inoculum was made by the method described above; molten cooled freshly prepared SDA medium was poured into the Petri plates. Keep the plates for about 30 min for solidification. Upon solidification, 200 µL of tested fungus spore suspension (1 × 10^6^ spores/mL) were spread on the SDA with the help of a sterile plastic L-rod spreader. The solutions of polyphenol compounds (Sigma Aldrich, extra pure ≥ 99%) were made by adding 1 mg of polyphenol compounds into the 1 mL of 10% DMSO. After vortex for 60 s, the sterile paper discs (6 mm in diameter) were dipped into 60 µL of the respective compound solution, and carefully kept into the inoculated agar plates. DMSO 10% added to the sterile paper disc was used as a negative control. The inoculated plates were kept in the incubator at 25 ± 2 °C for 36 h. Then the inhibition zone (mm) including the diameter of the disc was measured.

### Determination of the minimum inhibitory concentration (MIC) of key polyphenol compounds

The MIC of the identified key polyphenol compounds against the fruit rot fungus was determined in a 96-well plate with minor modification by the method as described by (Hemeg et al. 2020). Briefly, first of all, two-fold serial dilutions of polyphenol compounds were prepared into the 10% DMSO in concentrations ranging from 31.25 to 4000 µg/mL. From each well, 50 µL of each dilution was mixed with 200 µL of PDB (potato dextrose broth). In the negative control wells, 10% DMSO and PDB were added. Upon solidification, 10 µL of tested fungus spore suspension (1 × 10^6^ spores/mL) were added into the respective wells. The 96-well plates were then kept at 25 ± 2 °C for 7 d in the incubator. The MIC was considered the lowest concentration of polyphenol compounds which resulted in no visual growth of the respective fungus.

### Statistical analysis

The results of this study were presented as the mean value ± standard deviation (SD) and all the experiments were conducted in triplicate. The statistical evaluation was employed by analysis of variance, using SPSS (27.0, IBM Corporation, USA) statistical software. Tukey’s post hoc test following multi-comparative analysis of variance, or ANOVA, was applied to estimate the statistical significance of the treatment’s results (*P* < 0.05). The heatmap to compare LCMS results was plotted by using the website MetaboAnalyst 6.0 (https://www.metaboanalyst.ca/).

## Results

### Antifungal efficacy of pomegranate Peel extracts against postharvest fungi

**Fig. 2:**
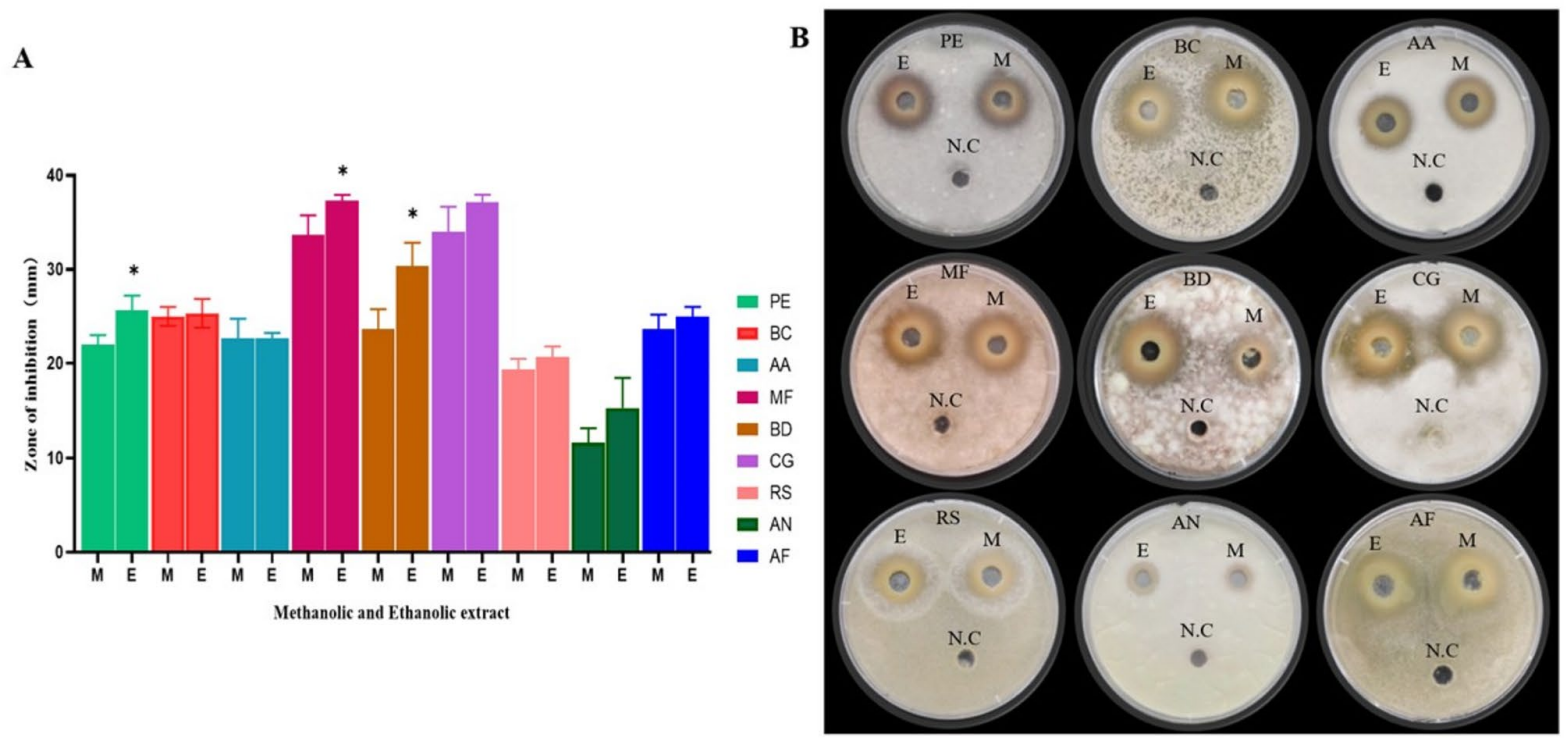
Inhibition zone of methanolic (M) and ethanolic (E) extracts of pomegranate peel against *P. expansum* (PE), *B. cinerea* (BC), *A. alternata* (AA), *M. fructicola* (MF), *B. dothidea* (BD), *C. gloeosporioides* (CG), *R. stolonifer* (RS), *A. niger* (AN), and* A. flavus* (AF). Asterisk (*) above the bar indicates a statistically significant difference (p< 0.05). The methanolic and ethanolic extracts labelled as M and E and negative control is represented by N.C

The antifungal activities of the methanolic and ethanolic extracts against various species of postharvest fungi were evaluated. Both the methanolic and ethanolic extracts significantly inhibited the growth of all tested fungi with different antifungal efficacy depending on the type of solvent used and species of fungi (Fig. 2). The greatest inhibition was observed on *C. gloeosporioides* and *M. fructicola* with ethanolic extract, while *(A) niger* showed the least growth inhibition for both methanolic and ethanolic extracts. The ethanolic extract generally showed greater antifungal activity across all fungal strains tested, of which the antifungal effects on *P. expansum*,* M. fructicola* and *B. dothidea* were significantly different (*P* < 0.05). Therefore, the ethanol crude extract was used in the following experiments.

**Fig. 3:**
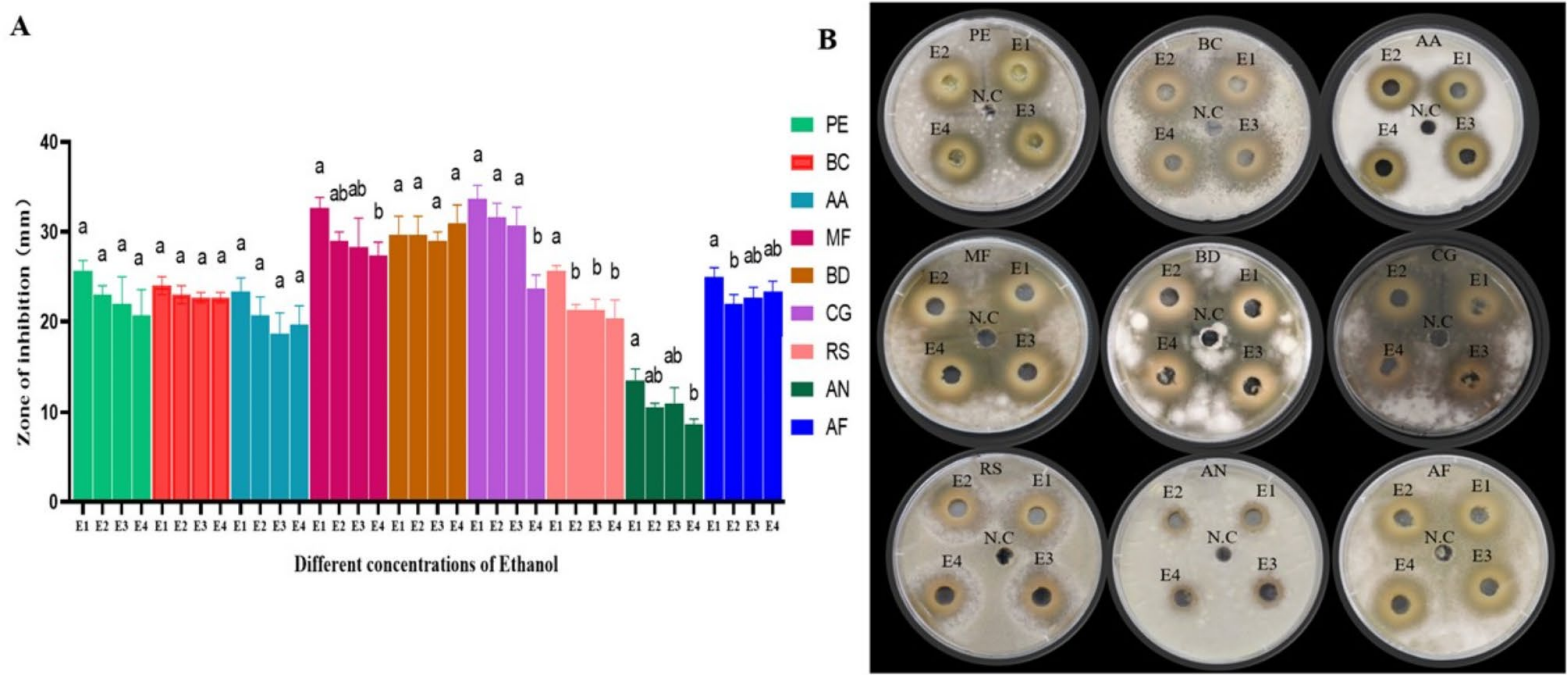
Inhibition zone of ethanolic 100% (E1), ethanolic 90% (E2), ethanolic 75% (E3) and ethanolic 50% (E4) extracts of pomegranate peel against *P. expansum* (PE), *B. cinerea* (BC), *A. alternata* (AA), *M. fructicola* (MF), *B. dothidea* (BD), *C. gloeosporioides* (CG), *R. stolonifer* (RS), *A. niger* (AN), and *A. flavus* (AF). The letters (a, b, c) specify statistical differences among different concentrations of ethanol, donating significant variations in antifungal activity. The ethanolic 100%, ethanolic 90%, ethanolic 75% and ethanolic 50% are labelled as E1, E2, E3 and E4, respectively and negative control is represented by N.C

When comparing the antifungal efficacy of pomegranate peel extracts prepared with different ethanol concentrations (100%, 90%, 75%, and 50%), it was observed that the 100% ethanolic extract exhibited the highest antifungal activity against all tested pathogens except *B. dothidea*. For *B. dothidea*, the 50% ethanol extract demonstrated the greatest inhibition zone, but there was no significant difference compared with other ethanol concentrations (Fig. 3A, B). All other fungi showed a declining trend of inhibition with the decrease of ethanol concentration, suggesting that higher ethanol concentrations are more effective for the extraction of the antifungal compounds from pomegranate peel.

### Antifungal efficacy, TPC, TFC of 3 fractions of ethanolic extract obtained via liquid-liquid partitioning

**Fig. 4:**
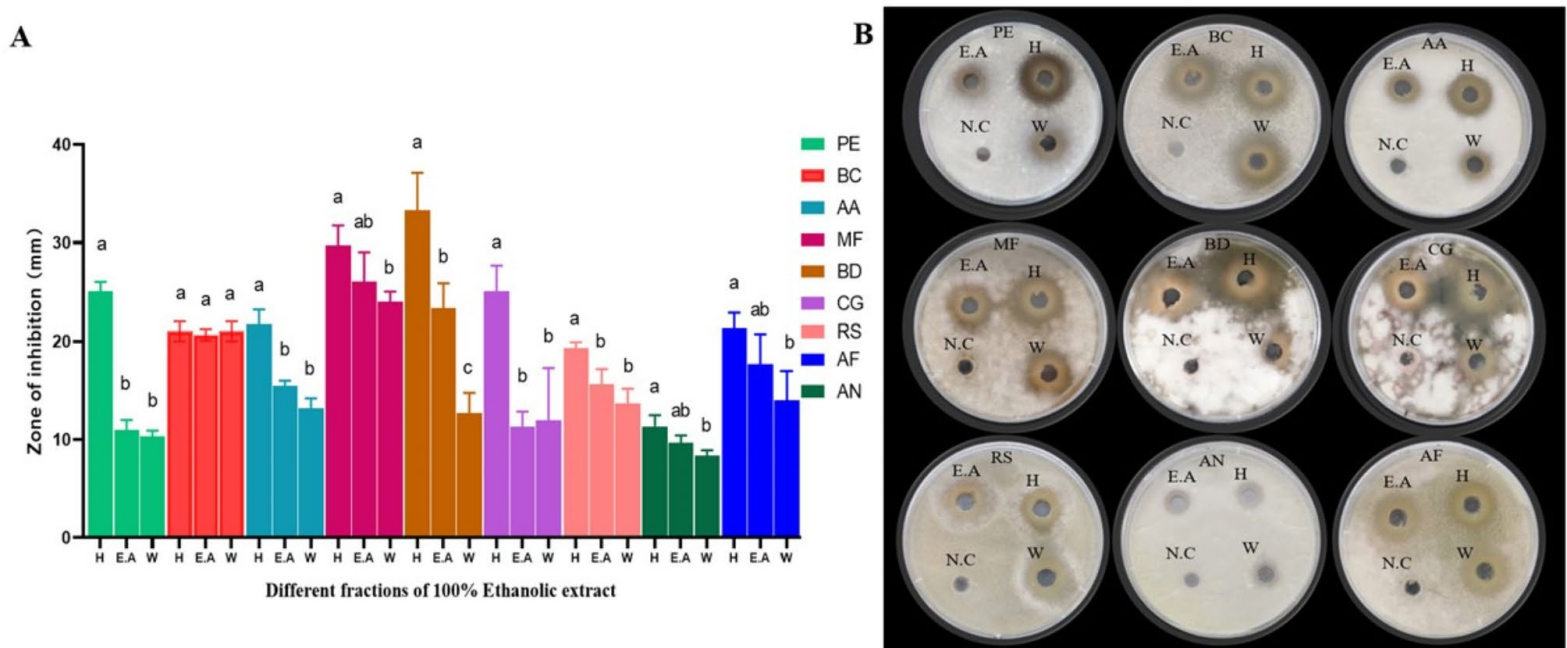
Inhibition zone of n-hexane fraction (H), ethyl acetate fraction (E.A) and water fraction (W) extracts against *P. expansum* (PE), *B. cinerea* (BC), *A. alternata *(AA), *M. fructicola* (MF),* B. dothidea* (BD), *C. gloeosporioides* (CG), *R. stolonifer* (RS), *A. niger* (AN), and *A. flavus* (AF). The letters (a, b, c) indicate statistical differences among fractions, presenting significant variations in antifungal activity. The n-hexane, ethyl acetate and water fractions are labelled as H, E.A and W, respectively and negative control is represented by N.C

Different levels of inhibition were observed when PPE fractions were tested against different fungal strains (Fig. 4). Except for *B. cinerea*, the n-hexane fraction showed the highest inhibition zones for all tested fungi compared to the ethyl acetate and water fractions. *B. cinerea* demonstrated a strong inhibition zone across all solvent fractions, representing consistent vulnerability to all tested solvent extracts. The maximum inhibition for the n-hexane fraction was observed against *B. dothidea* and *M. fructicola*. In contrast, *A. niger*, showed lower zones of inhibition across all 3 fractions, with n-hexane fraction still being the most effective compared to the aqueous and ethyl acetate fractions. These results indicated that the n-hexane fraction extract demonstrated the most pronounced antifungal effect (Fig. 4A, B), thereby being the most effective solvent for recovering antifungal compounds from pomegranate peel extract.

**Fig. 5:**
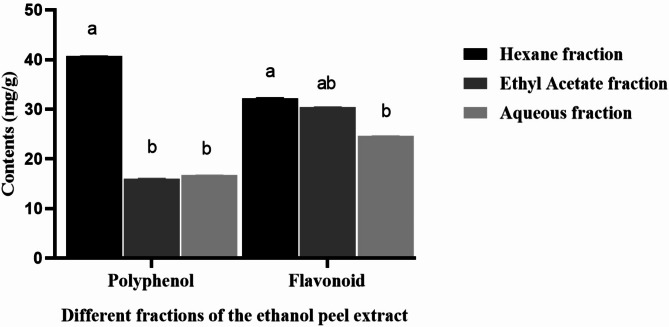
Total polyphenol and flavonoid contents in different fractions of the ethanol pomegranate peel extracts. Letters (a, b, c) highlight statistically significant differences at *p* < 0.05

Interestingly, the n-hexane fraction also showed higher TPC (40.70 ± 0.02 mg/g) and TFC (32.20 ± 0.11 mg/g) than the aqueous and ethyl acetate fractions (Fig. 5), indicating some correlations between polyphenols and the potent antifungal efficacy of the n-hexane fraction. Therefore, the polyphenol compounds in the pomegranate peel fractions were further examined in detail.

### Recognition of polyphenol compounds in pomegranate Peel fractions via UPLC-Q-TOF/MS

By using UPLC-Q-TOF/MS, a total number of 36 polyphenol compounds, including 6 phenolic acids and 30 flavonoids, were found across the 3 partition fractions of 100% ethanol extract. As listed in Table 1, the n-hexane fraction contained all 36 polyphenol compounds, whereas the water fraction contained only 27 of these compounds, with naringenin, quercetin, myricetin, caffeic acid, protocatechuic acid, luteolin-7-O-β-D-glucuronide, kaempferol, ophiopogonanone C, and salvianolic acid A being absent. The ethyl acetate fraction was in the absence of luteolin-7-O-β-D-glucuronide and salvianolic acid A, showing 34 polyphenol compounds.

**Table 1:** List of polyphenol compounds in different fractions of pomegranate Peel extract

Polyphenol	Molecular formula	Theoretical Mass (Da)	Experimental Mass (Da)	Error (ppm)	*N*-hexane fractiion	Ethyl acetate fraction	Water fraction
Hyperin	C_21_H_20_O_12_	463.08821	463.08844	0.5	+	+	+
Kaempferol-3-rutinoside	C_27_H_30_O_15_	593.15119	593.15177	1.0	+	+	+
Apigenin-7-glucoside	C_21_H_20_O_10_	431.09838	431.09834	-0.1	+	+	+
Phloridzin	C_21_H_24_O_10_	435.12966	435.13025	1.4	+	+	+
(-)-Epigallocatechin Gallate	C_22_H_18_O_11_	457.07762	457.07746	-0.4	+	+	+
Catechin	C_15_H_14_O_6_	289.07176	289.07079	-3.4	+	+	+
Guajavarin	C_20_H_18_O_11_	433.07764	433.07861	2.2	+	+	+
(-)-Catechin Gallate	C_22_H_18_O_10_	441.08272	441.08304	0.7	+	+	+
Naringenin	C_15_H_12_O_5_	271.06120	271.06117	-0.1	+	+	
Gallic acid	C_7_H_6_O_5_	169.01425	169.01436	0.7	+	+	+
(-)-Gallocatechin	C_15_H_14_O_7_	305.06667	305.06640	-0.9	+	+	+
Astragalin	C_21_H_20_O_11_	447.09329	447.09370	0.9	+	+	+
Quercetin	C_15_H_10_O_7_	301.03539	301.03526	-0.4	+	+	
Myricetin	C_15_H_10_O_8_	317.03029	317.03006	-0.7	+	+	
Ellagic Acid	C_14_H_6_O_8_	300.99898	300.99871	-0.9	+	+	+
Procyanidin B2	C_30_H_26_O_12_	577.13518	577.13521	0.1	+	+	+
Punicalin	C_48_H_28_O_30_	1083.05926	1083.0603	1.0	+	+	+
Punicalagin	C_34_H_22_O_22_	781.05300	781.05271	-0.4	+	+	+
Caffeic acid	C_9_H_8_O_4_	179.03498	179.03497	-0.1	+	+	
Protocatechuic acid	C_7_H_6_O_4_	153.01933	153.01943	0.7	+	+	
Luteolin-7-O-β-D-glucuronide	C_21_H_18_O_12_	461.07256	461.07236	-0.4	+		
Rutin	C_27_H_30_O_16_	609.14611	609.14637	0.4	+	+	+
Kaempferol	C_15_H_10_O_6_	285.04045	285.04044	0.0	+	+	
Kaempferol-3-gentiobioside	C_27_H_30_O_16_	609.14610	609.14637	0.4	+	+	+
Quercetin 3-O-β-D-galactopyranoside	C_21_H_20_O_12_	465.10275	465.10218	-1.2	+	+	+
Phloretin	C_15_H_14_O_5_	275.09140	275.09142	0.1	+	+	+
Genistin	C_21_H_20_O_10_	433.11292	433.11240	-1.2	+	+	+
Isoorientin	C_21_H_20_O_11_	449.10784	449.10713	-1.6	+	+	+
*p*-Coumaric acid	C_9_H_8_O_3_	165.05462	165.05477	0.9	+	+	+
Nobiletin	C_21_H_22_O_8_	403.13875	403.13806	-1.7	+	+	+
Ophiopogonanone C	C_19_H_16_O_7_	357.09688	357.09703	0.4	+	+	
Cinnamic acid	C_9_H_8_O_2_	149.05971	149.05964	-0.5	+	+	+
Apigenin	C_15_H_10_O_5_	271.06010	271.06026	0.6	+	+	+
Salvianolic acid A	C_26_H_22_O_10_	495.12858	495.13095	4.8	+		
Salidroside	C_14_H_20_O_7_	318.15474	318.15493	0.6	+	+	+
Syringin	C_17_H_24_O_9_	390.17586	390.17621	0.9	+	+	+

**Fig. 6:**
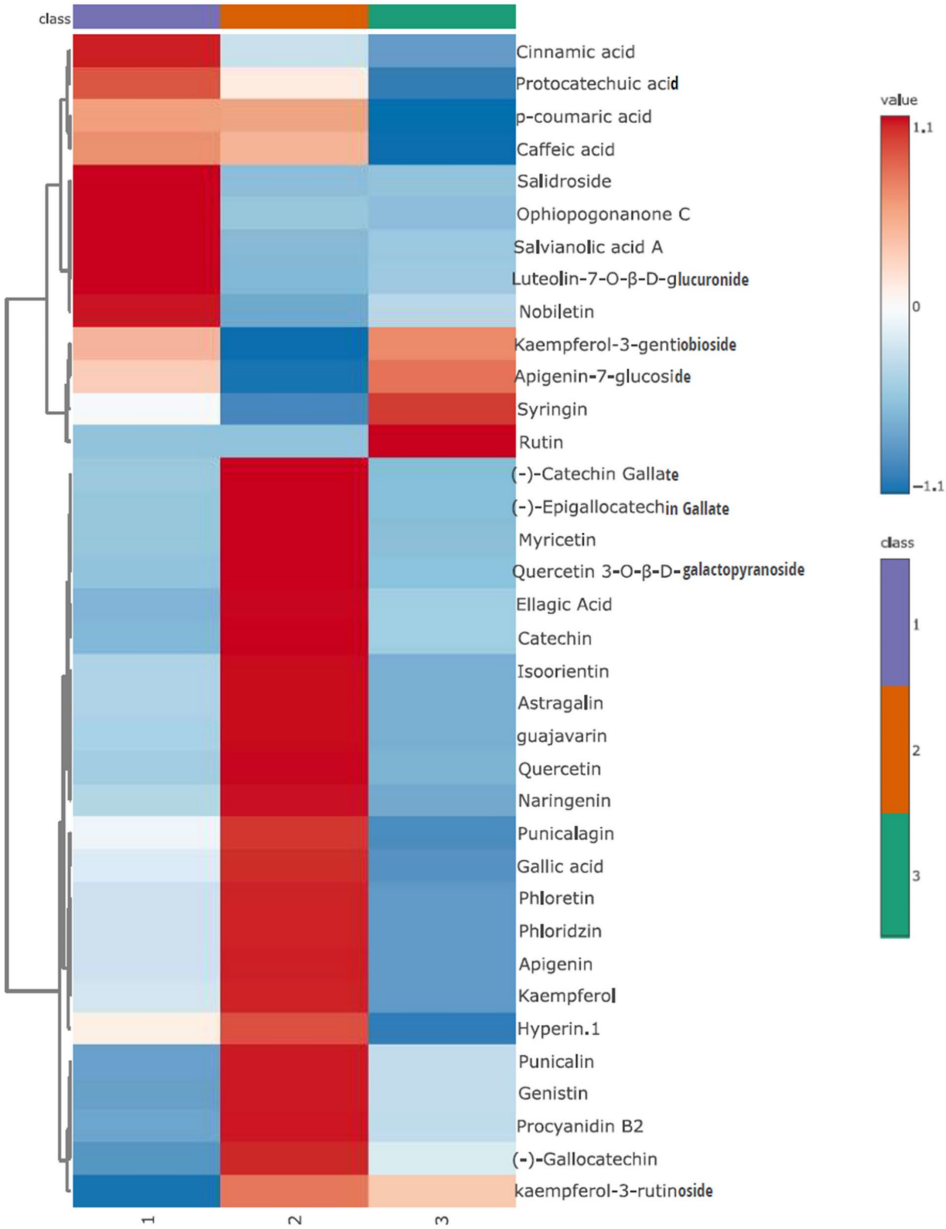
Heatmap to compare the polyphenol compounds in the three fractions (labeled on the bottom) of pomegranate peel extract. 1: n-hexane fraction; 2: ethyl acetate fraction; 3: water fraction. The brightness of the color indicates each compound’s concentration level in each sample. Red color represents the higher and blue color indicates lower levels of polyphenol compounds

A heatmap was created to elucidate the differences in the regulation and distribution of polyphenol compounds among the 3 partition fractions of pomegranate peel extract (Fig. 6). This visual representation effectively highlighted the varying relative concentrations of polyphenols across the 3 different solvent fractions. Results showed a comparative abundance of 9 specific polyphenols in the n-hexane fraction, including protocatechuic acid, caffeic acid, *p*-coumaric acid, cinnamic acid, salidroside, luteolin-7-O-β-D-glucuronide, salvianolic acid A, ophiopogonanone C and nobiletin. These 9 polyphenol compounds might play essential roles in the high antifungal activity of the n-hexane fraction of the pomegranate peel extract. Quantification results of these 9 specifically enriched polyphenols in the n-hexane fraction were shown in Table 2. Salidroside was the most abundant compound with a content of 48.65 ± 6.52 mg/g, followed by caffeic acid, protocatechuic acid and ophiopogonanone C, whereas salvianolic acid A was not detectable due to the lower level than the limit of detection (LOD) of the HPLC method.

**Table 2:** Content of 10 polyphenol compounds in n-hexane fraction and the Inhibition zone against different fungi

Compound	Content (mg/g)	PE (mm)	BC (mm)	AA (mm)	MF (mm)	BD (mm)	CG (mm)	RS (mm)	AN (mm)	AF (mm)
Protocatechuic Acid	1.69 ± 0.06^c^	12.5 ± 0.50^ab^	10.7 ± 1.53^ab^	11.0 ± 1.00^a^	13.0 ± 1.00^a^	11.2 ± 0.29^abc^	11.2 ± 0.29^ab^	11.3 ± 0.58^ab^	12.0 ± 1.73^ab^	11.3 ± 0.58^ab^
Salidroside	48.65 ± 6.52^b^	11.8 ± 0.76^ab^	10.7 ± 1.15^ab^	11.0 ± 0.50^a^	12.7 ± 0.29^ab^	11.7 ± 0.58 ^ab^	11.7 ± 0.58^ab^	11.7 ± 0.58^ab^	12.2 ± 0.76^ab^	11.7 ± 1.53^ab^
Caffeic Acid	3.73 ± 0.46^c^	12.0 ± 1.00^ab^	12.0 ± 1.00^a^	10.3 ± 1.53 ^a^	12.8 ± 0.29^a^	10.3 ± 1.53^cd^	10.3 ± 1.53^ab^	10.7 ± 0.58^b^	11.8 ± 1.04^ab^	12.0 ± 1.00^ab^
*p*-Coumaric Acid	0.47 ± 0.05^c^	12.7 ± 0.58^ab^	10.0 ± 1.00^b^	11.3 ± 0.58^a^	13.3 ± 1.15^a^	10.7 ± 0.58^bcd^	10.7 ± 0.58^ab^	11.3 ± 1.53^ab^	12.0 ± 1.00^ab^	11.7 ± 0.58^ab^
Luteolin-7-O-β-D-Glucuronide	0.13 ± 0.03^c^	11.8 ± 0.76^ab^	11.3 ± 0.58^ab^	11.0 ± 0.50 ^a^	12.5 ± 1.50^ab^	11.8 ± 0.29^ab^	11.8 ± 0.29^ab^	10.3 ± 1.15^b^	10.6 ± 0.58^b^	10.7 ± 0.58^b^
Cinnamic Acid	0.16 ± 0.00^c^	12.7 ± 1.15^ab^	11.7 ± 0.58^ab^	11.8 ± 1.04^a^	13.0 ± 1.00^a^	12.7 ± 0.29^a^	12.2 ± 0.29^a^	11.0 ± 1.00^ab^	12.0 = 0 ± 1.00^ab^	11.7 ± 0.58^ab^
Nobiletin	0.18 ± 0.01^c^	12.0 ± 1.00^ab^	11.7 ± 1.53^ab^	11.5 ± 1.00^a^	13.2 ± 0.29^a^	12.2 ± 0.76^a^	12.2 ± 0.76^a^	12.0 ± 1.00^a^	12.5 ± 0.87^a^	11.3 ± 1.53^ab^
Ophiopogonanone C	0.66 ± 0.07^c^	11.0 ± 1.73^b^	11.0 ± 1.00^ab^	10.0 ± 2.00^a^	10.7 ± 2.08^b^	9.8 ± 0.29 ^d^	9.8 ± 0.29^b^	10.5 ± 0.50^b^	11.7 ± 0.58^ab^	10.7 ± 1.15^b^
Punicalagin	70.66 ± 3.67^a^	13.0 ± 1.00^a^	11.7 ± 0.58^ab^	11.3 ± 0.58^a^	12.3 ± 1.15^ab^	11.0 ± 1.00^abcd^	11.0 ± 1.00^ab^	10.7 ± 0.58^b^	12.3 ± 0.58^ab^	11.7 ± 0.58^ab^
Ellagic acid	3.64 ± 0.55^c^	12.7 ± 1.15^ab^	11.3 ± 0.58^ab^	11.5 ± 0.50^a^	13.7 ± 1.15^a^	12.2 ± 0.29^a^	12.2 ± 0.29^a^	11.5 ± 1.32^ab^	11.83 ± 0.76^ab^	12.3 ± 0.58^ab^

Letters (a, b, c) in the same column presented statistically significant differences at *p* < 0.05. *P. expansum* (PE), *B. cinerea* (BC), *A*. *alternata* (AA), *M*. *fructicola* (MF), *B. dothidea* (BD), *C. gloeosporioides* (CG), *R. stolonifer* (RS), *A. niger* (AN), and *A. flavus* (AF)

Moreover, punicalagin (Brighenti et al. 2021) and ellagic acid (Rosas-Burgos et al. 2017) were reported as the key antifungal compounds in pomegranate peel extract. Therefore, the contents of these 2 compounds were also determined. Results showed the content of punicalagin (70.66 ± 3.67 mg/g) was higher than salidroside. The ellagic acid also showed a high content (3.64 ± 0.55 mg/g) in the n-hexane fraction, which is comparable to the content of caffeic acid.

### Antifungal efficacy of enriched polyphenol compounds in n-hexane fraction of PPE

The antifungal activity of the 8 accumulated and detected polyphenol compounds in the n-hexane fraction, along with punicalagin and ellagic acid, were tested by the disc diffusion method (Table 2). Results showed that various enriched polyphenol compounds in the n-hexane fraction of PPE demonstrated different inhibition zones against specific fungi. Among all the tested pathogens, the inhibition zones generally ranged between 9.8 ± 0.29 mm to 13.7 ± 1.15 mm. For example, *p*-coumaric acid showed the highest inhibition zone against *M. fructicola* and the least against *B. cinerea*. Ophiopogonanone C and luteolin-7-O-β-D-glucuronide showed comparatively smaller zones of inhibition for several fungi. The least inhibition zones against *B. dothidea*,* A. alternata*,* R. stolonifer* and *C. gloeosporioides* were found in ophiopogonanone C treatment, demonstrating weaker antifungal activity against these phytopathogens. Cinnamic acid exhibited higher zones of inhibition against multiple fungi, i.e. *M. fructicola*, *A. alternata*,* B. dothidea*, and *C. gloeosporioides*. Additionally, ellagic acid and punicalagin also displayed significant antifungal activity. Punicalagin showed the highest inhibition zone (13.0 ± 1.00 mm) against *P. expansum*. Nobiletin was demonstrated as the strongest antifungal agent against *C. gloeosporioides*,* R. stolonifer* and *A. niger*. Overall, the comparative antifungal activities of 10 polyphenol compounds against all 9 pathogens indicated that most compounds exhibited considerable inhibition against *M. fructicola*, whereas *B. cinerea* and *R. stolonifer* were the least affected across multiple compounds.

### Minimum inhibitory concentration (MIC) of enriched polyphenol compounds in n-hexane fraction of PPE

The MIC value of each polyphenol compound also varied against specific postharvest fungi (Table 3**)**. Protocatechuic acid displayed strong effectiveness against *A*. *alternata*,* R. stolonifer*, and *C. gloeosporioides* (MIC 250 µg/mL) with significant antifungal activity against *P. expansum* (MIC 125 µg/mL). The effectiveness of salidroside versus *P. expansum* was considerable (MIC 250 µg/mL), but its efficacy was limited against *R. stolonifer* (MIC 4000 µg/mL), *A. niger* and *A. flavus* (MIC > 4000 µg/mL). Caffeic acid was extremely potent against *P. expansum* (MIC 125 µg/mL). The antifungal activity of *p*-coumaric acid (MIC 250 µg/mL) toward *P. expansum*, *A*. *alternata*, *B. dothidea*, and *C. gloeosporioides*is was significant. Results also demonstrated that luteolin-7-O-β-D-glucuronide had little activity against *B*. *cinerea* (MIC 2000 µg/mL) while it displayed significant efficacy versus *P. expansum*, *B. dothidea*, and *C. gloeosporioides* (MIC 250 µg/mL). Cinnamic acid usually showed little antifungal activity against postharvest pathogens due to its high MIC values. Nobiletin showed considerable (MIC 250 µg/mL) activity against *M. fructicola*,* P. expansum*, and *B. dothidea*. Ophidroponanone C showed a low level of effectiveness against many fungi, including *P. expansum* (MIC 500 µg/mL). Furthermore, the MIC values of the ellagic acid were lower to intermediate level and it was more potent against *C. gloeosporioides*. Overall, the analysis of MIC values revealed that *B. dothidea*,* M. fructicola* and *C. gloeosporioides* were more sensitive fungi exhibiting comparatively lower MIC values for numerous compounds. In contrast, *R. stolonifera*,* A. flavus*,* and A. niger* were less affected, with constantly higher MIC values across several polyphenol compounds.

**Table 3:** MIC values (µg/mL) of the polyphenol compounds in n-hexane fraction against different fungi

Compound	PE	BC	AA	MF	BD	CG	RS	AN	AF
Protocatechuic Acid	125	1000	250	500	500	250	250	1000	500
Salidroside	250	1000	1000	500	500	500	4000	> 4000	> 4000
Caffeic Acid	125	1000	1000	500	1000	500	1000	500	250
*p*-Coumaric Acid	250	1000	250	500	250	250	> 4000	1000	1000
Luteolin-7-O-β-D-Glucuronide	250	2000	1000	500	250	250	500	500	1000
Cinnamic acid	1000	1000	1000	500	1000	250	1000	250	2000
Nobiletin	250	500	1000	250	250	500	> 4000	2000	2000
Ophiopogonanone C	500	1000	500	500	1000	4000	2000	1000	2000
Punicalagin	500	1000	250	500	250	250	500	500	250
Ellagic acid	1000	500	500	250	250	125	2000	500	500

PE (*P. expansum)*, BC (*B. cinerea)*, AA (*A*. *alternata)*, MF (*M*. *fructicola)*, BD (*B. dothidea)*, CG (*C. gloeosporioides)*, RS *(R. stolonifera)*, AN *(A. niger)*, and AF *(A. flavus).* The values are shown as mean ± standard deviations, and it is noted that standard deviation of zero was reported because three values are identical for cases, indicating no variability among those specific treatments

However, the efficacy results measured by the MIC method were not extremely consistent with the inhibition zones method. For example, salidroside exhibited an obvious inhibition zone of 12.2 ± 0.76 mm against *A. niger* but the MIC was greater than 4000 µg/mL. Similarly, caffeic acid had an MIC value of 1000 µg/mL against *B. cinerea* with inhibition diameters of 12.0 ± 1.00 mm. Punicalagin showed a significant inhibition zone via *P. expansum*, *M. fructicola* and *A. niger* (13.0, 12.3, and 12.3 mm, respectively), yet the MIC values were high at 500 µg/mL. The different results might be due to the different polar and concentrations of compounds, the medium’s composition, incubation time and environmental factors (Eloff 2019).

## Discussion

### Effective extracts with higher antifungal activity from pomegranate Peel

The ethanolic extracts were more effective than methanolic extracts against fungi like *P. expansum*,* B. cinerea*,* A. alternata*,* M. fructicola*,* B. dothidea*, *C. gloeosporioides*, *R. stolonifer*,* A. niger*,* and A. Flavus*, with the highest inhibition against *M. fructicola***(**Fig. 2**)**. It was also interesting to note that, 100% ethanolic extract demonstrated the strongest inhibitory zone against most of the fungi, in comparison to other concentrations **(**Fig. 3**)**. Similar results were reported that the ethanol extract of Jordanian pomegranate peel showed higher efficacy against *Micrococcus luteus* compared to methanol extract (Sweidan et al. 2023). Ethanol extract also demonstrated the highest antimicrobial effects against various microbial strains (Ibrahium 2010). Ethanol was chosen as the extraction solvent due to its favourable environmental and safety profile. As a biodegradable and non-toxic solvent approved for use in pharmaceuticals and food processing, it is a preferable choice than methanol (Fang et al. 2025). These advantages make it particularly suitable for postharvest biocontrol applications. Moreover, these properties not only align with green chemistry principles but also enhance the overall sustainability and safety of the extraction process, making the resulting PPE-derived antifungal agents suitable for potential use in food systems.

Ulteriorly, the antifungal activity of different partitioned fractional extractions of the ethanolic extract was investigated. N-hexane fractions showed the greatest efficacy **(**Fig. 4**)**. This could be attributed to the higher concentration of antifungal compounds in the n-hexane fraction with the highest total phenol content **(**Fig. 5**)**. Phenolic compounds had been confirmed to possess significant antimicrobial activity (Wang et al. 2017; Hernández et al. 2021; Pane et al. 2016). Higher levels of polyphenols associated with effective antimicrobial activities might be due to the forming complexes with microbial cell wall proteins and leading to cell lysis (Akhtar et al. 2015; Dey et al. 2012; Sorrenti et al. 2019). It was also reported the n-hexane fraction was the one with the highest polyphenol concentration from PTO8 cultivar pomegranate peel (Rosas-Burgos et al. 2017). The solvent commonly affected the extraction of different phenolic compounds. N-hexane fraction might contain several compounds with strong antifungal activity which were enriched in this partition. However, previous research indicated the butanol fraction was the most effective for inhibiting fungal growth (Rosas-Burgos et al. 2017). This difference might result from the pomegranate cultivar containing antifungal compounds other than those commonly reported in peel extracts (ellagic acid and punicalagin).

### Phenolic compounds in pomegranate Peel extract with specific antifungal activity

The UPLC-Q-TOF/MS analysis detected 36 distinct polyphenol compounds in pomegranate peel extract **(**Table 1**)**. Several studies have successfully employed LC-MS and its variants (including UPLC-Q-TOF/MS) for the analysis and identification of phenolic compounds, indicating its reliability and capability for this purpose (Yang et al. 2024; Qiu et al. 2024; Teng et al. 2022; Zhang et al. 2021). Various studies have identified phenolic compounds in pomegranate peels, including rutin, tiliroside, apigenin-7-glucoside, gallocatechin, catechin, epigallocatechin gallate, ellagic acid, gallic acid, punicalagin, punicalin and procyanidin B2 (Elkahoui et al. 2024; Jebahi et al. 2022; Abdulla et al. 2017; Ambigaipalan et al. 2016; El-Hadary and Ramadan 2019). The variations in polyphenols detected could be influenced by the measurement method, gradient, apparatus, mobile phase, and extraction solution (Man et al. 2023). To our best knowledge, salidroside, ophiopogonanone C, nobiletin, sophoricoside, isoorientin, quercetin 3-O-β-D-galactopyranoside, kaempferol-3-gentiobioside, luteolin-7-O-β-D-glucuronide, guajavarin, and catechin gallate, were not reported in previous studies. These 10 compounds are newly recognized polyphenols in the pomegranate peel.

In order to find out the main polyphenol compounds contributing to the effective antifungal activities in pomegranate peel extract, 8 comparative abundance polyphenols in n-hexane partitioned fraction and 2 notable polyphenol compounds (punicalagin and ellagic acid) were quantified and their antifungal activities were evaluated. In particular, salidroside, a newly discovered phenolic compound in pomegranate peel extract, showed a quite high amount in the n-hexane fraction. It was reported that salidroside isolated from the orange peel possessed antifungal activity and significantly inhibited the growth of postharvest fungi up to 30% (Hernández et al. 2021). Another study (Zaushintsena et al. 2020) also reported that the salidroside (isolated from *Rhodiola rosea* L.) had strong antimicrobial activity against the coccoid and rod bacteria. The salidroside might be an essential polyphenol compound in pomegranate peels and should gain more attention in research on pomegranate peel bioactivities.

Earlier research had demonstrated a correlation between the amount of phenolic acids in polyphenolic plant-derived extracts and their antifungal efficacy (Wang et al. 2017; Hernández et al. 2021; Pane et al. 2016). In this study, phenolic acids such as *p*-coumaric acid, protocatechuic acid, caffeic acid, ellagic acid, and cinnamic acid showed the highest antifungal activity (low MIC value) across the multiple fungus strains depending on the target fungi and media composition. The MIC value of *p*-coumaric acid was 250 µg/mL toward *P. expansum*, *A. alternata*, *B. dothidea*, and *C. gloeosporioides*. A previous study reported that *p*-coumaric acid from orange peel significantly inhibited the growth of *A. alternata*, *B. cinerea*, and *M. fructicola* in synthetic media (Hernández et al. 2021). These discoveries supported *p*-coumaric acid’s potential as a broad-spectrum antifungal agent, making it suitable for controlling postharvest fungal diseases. Ellagic acid, known for its high antioxidant potential, also demonstrated strong inhibition against *C. gloeosporioides* and *M. fructicola*, aligning with previous research highlighting its antimicrobial properties in the pomegranate peel extract (Rosas-Burgos et al. 2017). Protocatechuic acid exhibited significant efficacy against *P. expansum*,* C. gloeosporioides*,* R. stolonifera* and *A. alternata*, while cinnamic acid showed strong antifungal activity against *C. gloeosporioides* and *A. niger*, consistent with its well-documented antioxidant and antifungal properties (He et al. 2023).

The exact mode of action of phenolic acids is not fully understood and it has been proposed that their lipophilic features and hydroxyl group enable them to alter the functionality of biological membranes (Lattanzio et al. 1994). According to Morales et al. (2017), p-coumaric acid may inhibit *B. cinerea* by uncoupling oxidative phosphorylation, allowing the compound to pass through cellular membranes without damaging their integrity. Furthermore, Lou et al. (2012) investigated the antibacterial mechanism of *p*-coumaric acid and concluded that it triggered potassium ion efflux from living cells, indicating a rise in membrane permeability. Their study results also revealed that *p*-coumaric acid binds to DNA, potentially altering its composition, inducing membrane damage, and leading to cell death (Lou et al. 2012). Similarly, caffeic acid, due to its nucleophilic properties, could give a pair of electrons to membrane lipids and proteins, breaking membrane integrity and allowing internal elements to flow out and then change the activities of cells (Perumal et al. 2017). These findings suggested that the antifungal effects of pomegranate peel phenolic acids were likely mediated through different multiple mechanisms, including membrane disruption, interference with cellular processes, and perhaps DNA binding. By targeting and altering these cellular functions essential for fungal survival and growth, these phenolic acids effectively inhibit postharvest fungal pathogens.

Other non-phenolic acid compounds in the n-hexane extract, such as nobiletin, luteolin-7-O-β-D-glucuronide and punicalagin also exhibited antifungal activity. Previous studies highlighted the higher contents of the punicalagin contributing significantly to the antifungal properties of pomegranate peel extract (Glazer et al. 2012; Brighenti et al. 2021). Tannins (punicalagin) may affect microbes by disrupting their metabolism, changing their cell membrane composition, making complexing with essential metallic ions essential for metabolism, and preventing bacterial and fungal enzymes from interacting with their substrates (Aldarhami et al. 2023; Noumi et al. 2022; Kulkarni et al. 2007). Comparative results showed that the compound ophiopogonanone C had a lower inhibition zone across various fungi.

The findings of this article highlighted that the antifungal activity of pomegranate peel extract was largely driven by its higher contents of polyphenols, which likely contributed synergistically to its effectiveness against various fungal pathogens. The majority of previous investigations commonly focused on the antimicrobial effects of ellagic acid and punicalagin, with very little consideration given to other polyphenols in the peel extracts. This study showed that n-hexane fraction and its polyphenol compounds demonstrated high inhibition activity against the specific fungi. To the best of our knowledge, this is the first report on the antifungal activity of pomegranate peel extract against *B. dothidea* and *M. fructicola.* Additionally, various polyphenol compounds in pomegranate peel except for punicalagin and ellagic acid were examined for the first time against different postharvest fungal pathogens. However, the antifungal mechanism as well as the evaluation of polyphenol efficacy and stability under real-world postharvest environments, were beyond the scope of this study. Future research should focus on assessing the efficacy of pomegranate peel polyphenols over time and across diverse storage conditions to validate their practical applicability and optimize their use in real-world scenarios. The detailed antifungal mechanisms at the biochemical and molecular-lever, along with potential interactions with fruit physiological resistance, should be explored in subsequent studies. These findings will further underscore the potential of pomegranate peel polyphenols for postharvest disease management and lay the foundation for their effective application in fruit preservation practices.

## Conclusions

The results of this study concluded that the n-hexane fractions of the pomegranate peel ethanolic extract showed the highest inhibitory activity on the growth of all tested postharvest fungus pathogens with higher levels of polyphenols and flavonoid concentration. Totally 36 polyphenol compounds in pomegranate peel extract were detected and 9 of them showed a comparative abundance in the n-hexane fraction. Salidroside and nobiletin, which were newly identified in pomegranate peel and enriched in hexane fractions showed significant antifungal activity, especially against *P. expansum*, *M. fructicola*,* B. dothidea* and *C. gloeosporioides*. Other polyphenol compounds enriched in hexane fraction, including protocatechuic acid, caffeic acid, *p*-coumaric acid and cinnamic acid, also possessed significant antifungal activity against different fungi. The results raised the possibility of the use of these compounds in the pomegranate peel extracts as natural antifungal or antimicrobial agents and paved the way for the development of natural, sustainable preservation techniques to reduce fruit postharvest losses. Future research should prioritize the development of commercially viable formulations of pomegranate peel extracts (PPE), focusing on stability optimization and efficient application systems. Establishing standardized protocols for concentration, application timing and treatment frequency will be essential to bridge the gap between laboratory-scale findings and large-scale agricultural implementation. Additionally, its potential synergistic effects with other natural antimicrobials and physical preservation techniques under real-world field conditions also need to be investigated.

## Electronic supplementary material

Below is the link to the electronic supplementary material.

Supplementary Material 1

## Data Availability

The data are available from the corresponding author on reasonable request.
