# Supplementary figures and images for: Integrated evaluation of antifungal activity of pomegranate peel polyphenols against a diverse range of postharvest fruit pathogens
Source: Bioresour Bioprocess. 2025 Apr 15;12(1):34. doi: 10.1186/s40643-025-00874-9 (PMC11996745; doi:10.1186/s40643-025-00874-9)

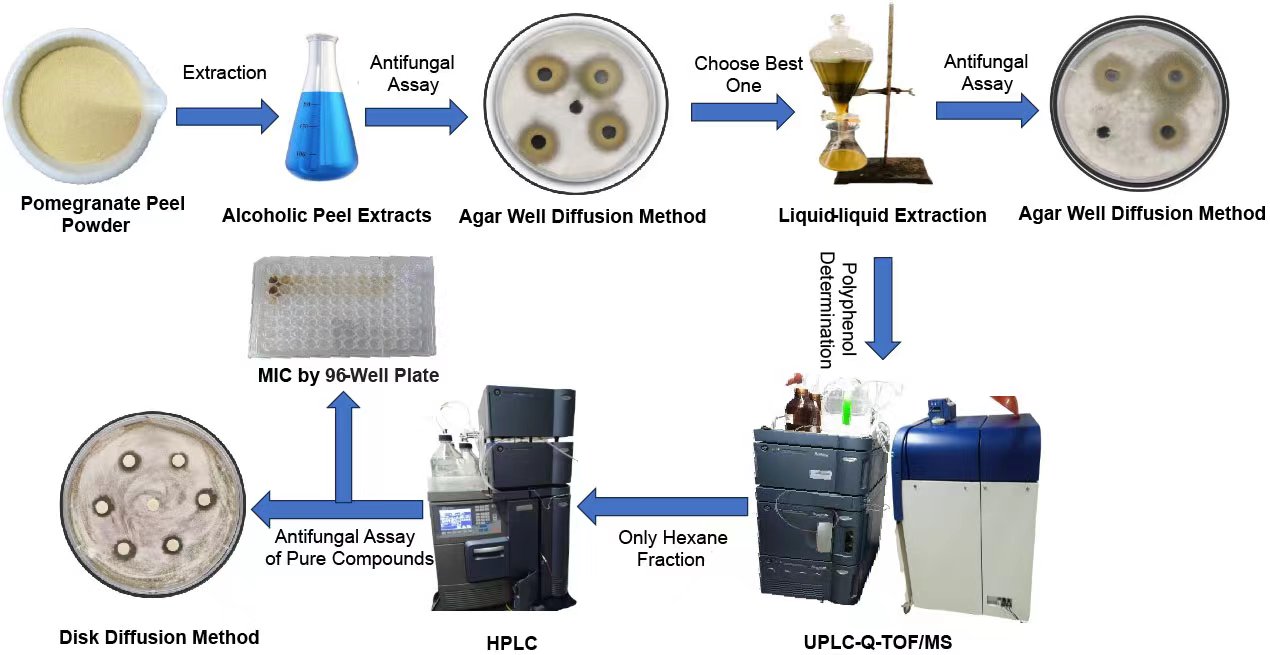

Supplement: Supplementary file 1 — Supplementary Material 1 [file 40643_2025_874_MOESM1_ESM.jpg]
